# Closed versus open reduction of facial fractures in children and adolescents: A systematic review and meta-analysis

**DOI:** 10.4317/jced.57323

**Published:** 2021-01-01

**Authors:** Igor Pereira, Eduardo Pellizzer, Cleidiel Lemos, Sandra Moraes, Belmiro Vasconcelos

**Affiliations:** 1 Department of Prosthodontics and Bucco Facial Surgery, University of Pernambuco. Brazil

## Abstract

**Background:**

Treatment of facial fractures in children and adolescents has always been a challenge for oral surgeon. The choice of treatment type must take into account several factors. This systematic review aimed to evaluate closed versus open reduction of facial fractures for pediatric facial fractures.

**Material and Methods:**

A systematic review of the literature was conducted in three databases (PubMed/MEDLINE, Embase and The Cochrane Library) in accordance with the PRISMA statement. The PICO question was: Conservative treatment is more appropriate than surgical treatment for reducing facial fractures in children and adolescents? The full papers of 41 references were analyzed in detail. Eleven papers were included in this systematic review: one prospective study and ten retrospective studies. All studies evaluated the complication rate.

**Results:**

A total of 73 (7.68%) of the 950 patients experienced complications. Among these patients, 24 (3.85%) had been treated with conservative treatment and 49 (15.03%) with surgical treatment. The fixed-effects model revealed a lower complication rate with conservative treatment than surgical treatment (*P*<0.00001; RR: 0.18; 95% CI: 0.11–0.28). Heterogeneity was low for the complication rate outcome (X2: 5.64; *P* = 0.69; I2: 0%).

**Conclusions:**

The present findings show that conservative treatment is more commonly performed for pediatric facial fractures and complications occur more with surgical treatment. Therefore, surgeons must evaluate all variables involved in choosing the most appropriate treatment method to ensure greater benefits to the patient with fewer complications.

** Key words:**Closed fracture reduction, open fracture reduction, pediatrics, treatment failure.

## Introduction

Facial fractures in children are relatively rare and evaluated separately due to their particular diagnostic and treatment aspects. In children, bones have greater elasticity and there is less pneumatization of the sinuses, greater thickness of the surrounding adipose tissue and good stability of the maxilla and mandible due to the presence of unerupted teeth. Due to these characteristics, considerable energy is required to cause a fracture in developing bones (1). The prevalence of facial fractures in children and adolescents is approximately 10%. The majority of fractures occur past the age of five years, with peaks of incidence at school age and in adolescence, when the characteristics of craniofacial traumas are similar to those found in adults (2).

Social, cultural and environmental factors are responsible for altering the epidemiology of craniofacial trauma. The incidence of facial fractures in the pediatric population is higher among boys at almost all ages, with a ratio of up to 3:1 in comparison to girls (3,4).

Divergent opinions are found in the literature regarding the treatment of facial fractures in pediatric patients, but there is a consensus that changes in growth should be prevented and more conservative treatment (non-surgical) is indicated, whenever possible (5). In many cases, however, it is necessary to perform open fracture reduction, for which an absorbable fixation system or titanium miniplates are commonly used (6).

Within this context, the aim of the present study was to perform a systematic review of the literature to evaluate closed versus open reduction of facial fractures for pediatric facial fractures.

## Material and Methods

-Registry protocol

This systematic review was structured following the PRISMA checklist (7) and was performed in accordance with models proposed in the literature (8,9). The methods used in this systematic review are registered with the international prospective register of systematic reviews (PROSPERO: CRD42018094847).

Search strategy and information sources

Two independent reviewers (CAAL and CCM) performed the article selection process using pre-established eligibility criteria. Studies were pre-selected on the basis of the titles and abstracts and assessed according to the inclusion and exclusion criteria. The reviewers analyzed and discussed the articles until a consensus was reached. Any disagreements were resolved through discussions with a third reviewer (BCEV).

The following databases were searched for the identification of relevant articles: PubMed (http://www.ncbi.nlm.nih.gov/pubmed), Web of Science (http://appswebofknowledge.ez27.periodicos.capes.gov.br/WOS_GeneralSearch_input.do?product=WOS&search_mode=GeneralSearch&SID=6AgXsKu6D9IhbLBoyku&preferencesSaved=) and The Cochrane Library (http://onlinelibrary.wiley.com/ cochranelibrary/search/). The following keywords were used: ((((pediatric OR children OR adolescents OR child OR paediatric)) AND (facial trauma OR facial fracture OR maxillofacial fracture OR maxillofacial trauma OR mandibular fracture OR mandibular trauma OR midface)) AND (Open reduction OR Miniplate OR screw devices OR Titanium plate OR Resorbable plate OR internal fixation OR ORIF OR osteosynthesis)) AND (Conservative OR closed reduction OR immobilization OR Arch bar OR Close observation OR non-invasive treatment OR IMF).

-Selection criteria

The inclusion criteria for the initial selection were publications in English with no restriction imposed on the date of publication, studies involving human subjects, specific studies on treatment for facial fractures in children and adolescents and descriptions of the number of patients treated, proposed treatment (surgical access and osteosynthesis materials), postoperative characteristics, complications, follow up and conclusions. After the pre-selected articles had been submitted to full-text analysis, the criteria listed in Table 1, were used for the final selection of papers for inclusion in the present review. The selection criteria were established by the authors prior to the onset of the study.

**Table 1:**
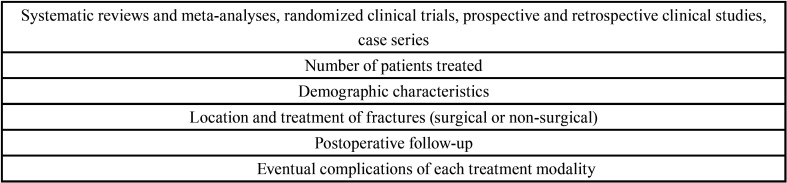
Eligibility criteria.

An inter-examiner test (kappa) was performed to determine the level of agreement regarding the pre-selection of studies based on the titles and abstracts. The following kappa values were found for the different databases: PubMed/MEDLINE: 0.83; Embase: 0.80; Cochrane: 1.0.

-Criteria for the selection of studies

The first phase of the article selection process was analysis of the titles and abstracts of the papers retrieved during the searches of the databases. Articles having passed this first step were submitted to full-text analysis based on the eligibility criteria. The PICO question recommended in the PRISMA statement was determined as follows: (P) Population: children and adolescents with facial fracture; (I) Intervention: open treatment (surgery); (C) Comparison: closed (conservative) treatment; (O) Outcome: complications (inflammatory process; facial growth). The following was the guiding question: Conservative treatment is more appropriate than surgical treatment for reducing facial fractures in children and adolescents?

-Exclusion criteria

The following were the exclusion criteria: *in vitro* studies, animal studies, reviews, case reports, case series, studies in which complications are not reported, oral communications, posters and studies that do not report the type of treatment performed to reduce fractures.

-Analysis of methodological quality

The methodological quality of the studies was assessed independently by the same two investigators. The materials and methods, results and discussion sections were analyzed using the Cochrane Collaboration tool for assessing the risk of bias.

The quality of the selected studies was evaluated based on the PRISMA criteria, using the 27 questions established by Moher *et al*. (7). Therefore, the studies were separated into categories of randomized clinical trials and prospective studies.

-Meta-analysis 

The Reviewer Manager 5 (Cochrane Group) software program was used for the meta-analysis, which was based on the Mantel-Haenzel (MH) method. The dichotomous outcome (complication rate) was analyzed using risk ratios (RR) and respective 95% confidence intervals (CI). Data were considered significant when *P* < 0.05. In cases of statistically significant heterogeneity (*P* < 0.10), a random-effects model was used, whereas a fixed-effects model was used in cases of a non-significance difference (10). A funnel plot (plot of effect size versus standard error) was created to evaluate the occurrence of publication bias.

## Results

-Search results

**Figure 1:**
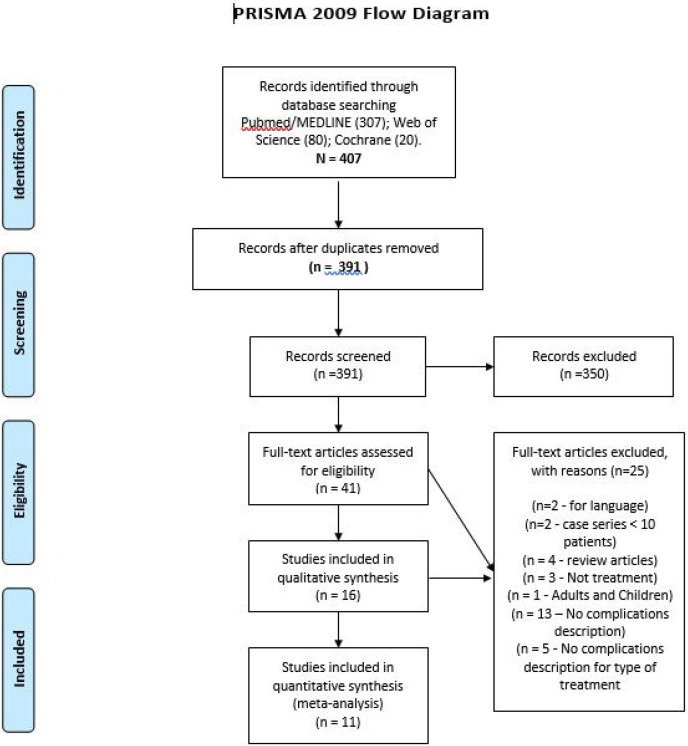
QUORUM flow diagram of the article selection process.

The electronic searches were performed in April 2018 and yielded 307 references in PubMed, 80 in Embase, and 20 in The Cochrane Library. No additional studies were identified in the manual searches. After the removal of duplicates, 391 potentially relevant references were assessed, 41 of which were submitted to full-text analysis. The application of the eligibility criteria led to the exclusion of thirty articles. Thus, eleven articles were found to be clinically or technically relevant to the subject of the study and were included in this systematic review. The AQUORUM flow diagram giving an overview of the selection process is presented in Fig. 1.

-Types of studies

Among the eleven papers included in this systematic review, ten were retrospective studies (11-20) and one was a prospective study (21). Table 2 displays the characteristics of these studies, that were divided by region of the affected face, mandibular condyle (11,13,20), mandible (12,16,17,19,21) and all regions of the face (14,15,18).

**Table 2:**
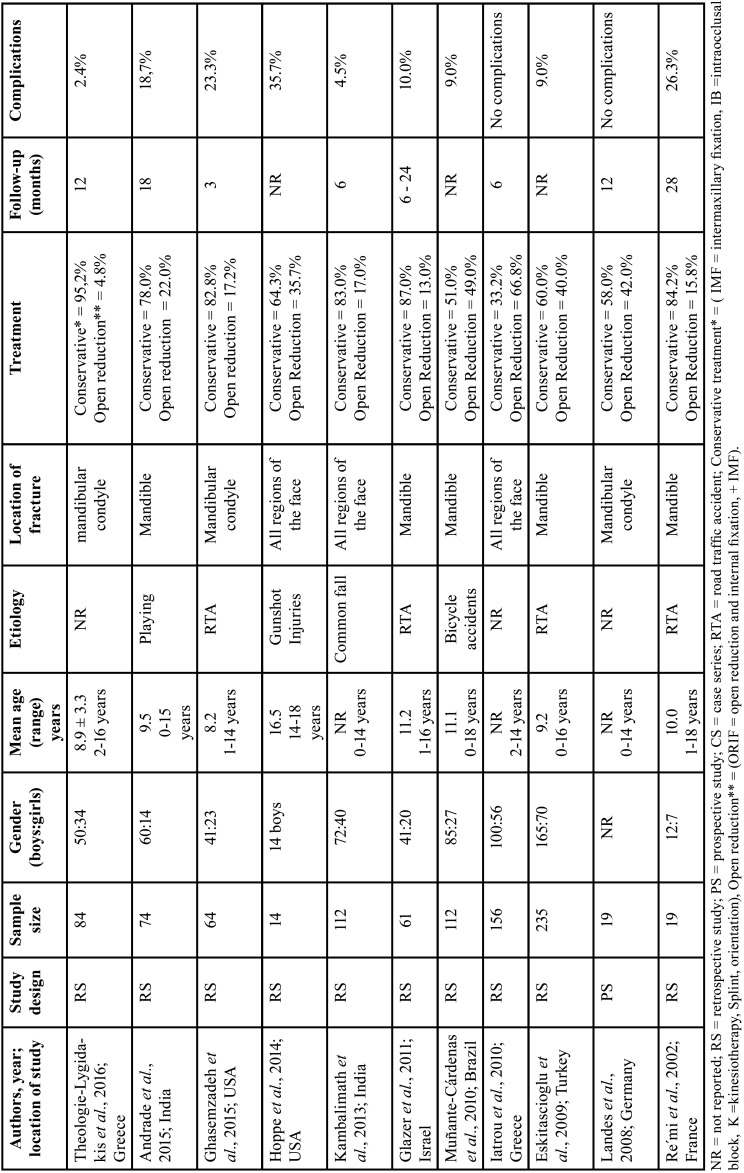
Studies included in the systematic review.

The Cochrane Collaboration tool for assessing the risk of bias could not be applied due to the type of studies included in this systematic review. Consequently, the Newcastle-Ottawa scale (NOS) for assessing the quality of non-randomized studies was used (Table 3).

**Table 3:**
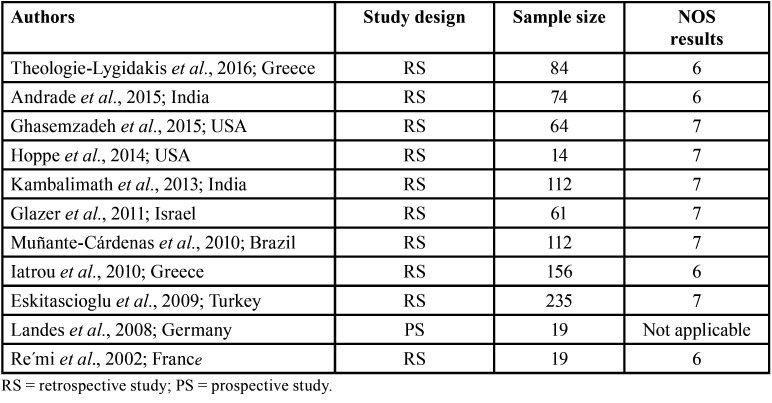
Newcastle–Ottawa quality assessment scale (NOS): range from 0 to 9.

-Meta-analysis 

**Figure 2:**
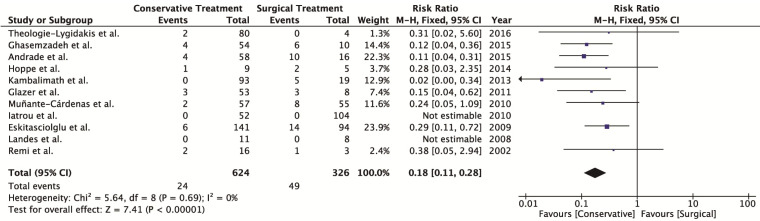
Forest plot of heterogeneity for type of treatment and complications rate outcome.

**Figure 3:**
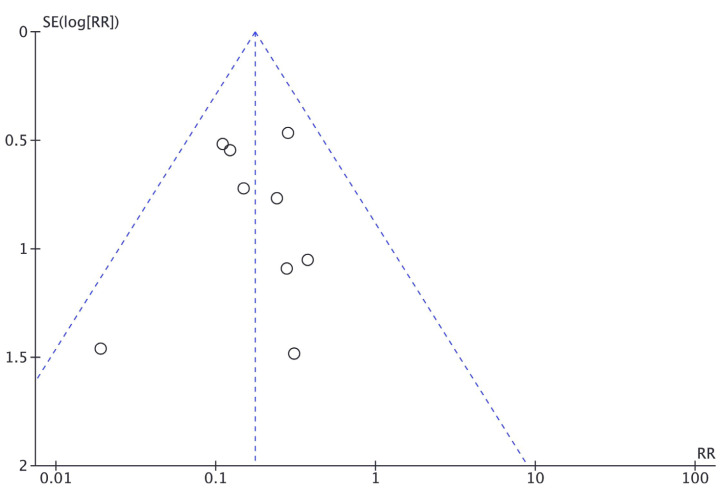
Funnel with symmetry of included studies for complications rate.

All studies evaluated the complication rate. A total of 73 (7.68%) of the 950 patients had complications. Among these patients, 24 (3.85%) had been treated with conservative treatment and 49 (15.03%) had been treated with surgical treatment. Two studies reported no complications in either group evaluated. The fixed-effects model revealed a significantly lower complication rate with conservative treatment compared to surgical treatment (*P* < 0.00001; RR: 0.18; 95% CI: 0.11 to 0.28). Heterogeneity was considered low for the complication rate outcome (X2: 5.64; *P* = 0.69; I2: 0%) (Fig. 2). The funnel plot demonstrated symmetry in the studies with regard to the complication rate, suggesting an absence of publication bias (Fig. 3).

## Discussion

The issue of facial fractures in children and adolescents is important. However, the difficulty in managing these patients imposes limitations on the types of study conducted to address this subject. Thus, there is an absence of clinical trials due to the required sample size and the variety of types of treatment (conservative or non-surgical and surgical) (6,22).

In the studies analyzed, mean age was 10.2 years, demonstrating a greater occurrence of facial trauma in adolescence, as reported previously (23,24). This type of trauma generally occurs as a child becomes more independent from family life and has greater contact with contact sports, urban violence or physical aggression at school or on the street (2,25,26).

In nearly all studies, the prevalence of facial trauma was higher in the male sex (11-19,21). This trend is also observed in studies involving adults, as males in all age groups are more exposed to violence and accidents, such as traffic accidents at an older age and domestic violence at a younger age (24,27).

The present study divided the fractures of the face by affected bone to facilitate the analysis and understanding, so that it is possible to show the differences between them, regarding the type of treatment and the complications, for example.

In relation to the etiology of trauma, traffic accidents were the most prevalent (13,16,19,21). While this cause has declined due to laws requiring seat belts and car seats for children, such precautions are often neglected, leaving children unprotected and exposed in the event of an accident (28,29).

The literature reports that the mandible is the most affected in children and adolescents (30-31). In the present review, eight studies (11-13,16,17,19-21) reported cases of mandibular fracture, which may be explained precisely by this high prevalence rate. Moreover, the treatment of this type of fracture poses a challenge in both children and adults, with different forms of conservative and surgical treatment proposed, depending on the affected region of the mandible (32,33).

Regarding the type of treatment, conservative methods were more commonly employed, regardless of age, although it has been reported that conservative treatment is generally used for younger children, depending on the energy and location of the trauma (34,35). Open treatment is generally performed with rigid internal fixation, especially titanium plates (36,37).

Among the studies analyzed, the most prevalent conservative treatment was intermaxillary fixation for a period of two weeks, which is in agreement with data reported previously (11,15). However, other forms of conservative treatment were also used, such as intraocclusal block (12,13,19), kinesiotherapy (11,21), splint (16) and orientation (14,17). However, Neff *et al.* (38) reported that surgical treatment for facial fractures in children has been increasingly more frequent in recent years, especially as children get older.

The difference between treatments with regard to complications was significant, with a lower prevalence found for conservative treatment (12-17,19). This finding was expected, as the possibility of complications in open treatments is inherently greater due to the use of fixation materials and the risk of infection or nerve injury (6). The complications following conservative treatment were generally related to small asymmetries or deviations, which are expected in certain types of trauma (11,19).

In cases of conservative treatment for condylar fractures in children, complications such as temporomandibular disorder and ankylosis of the temporomandibular joint (39) may be observed years after the fracture. However, as the follow up of these patients is limited, many of these complications are not reported. With surgical treatment for this type of fracture, the complications are generally related to nerve function, especially the temporal branches, zygomatic branches of the facial nerve and the auriculotemporal nerve (37). The fixed effects model was performed with nine articles, since two reported no complications, and revealed a significantly lower rate of complications with conservative treatment compared to surgical treatment. Moreover, the funnel plot demonstrated symmetry among the studies regarding the complication rate, indicating an absence of publication bias.

This systematic review included studies that evaluated the treatment of facial fractures in children and adolescents, but not all the studies were used in the meta-analysis due to methodological heterogeneity, which was mainly related to the complications resulting from treatment, thereby limiting the information available on these outcomes.

The present findings show that conservative treatment is more commonly performed for pediatric facial fractures and, as demonstrated by the meta-analysis, leads to a significantly lower occurrence of complications when compared to surgical treatment. The most common forms of conservative treatment are intermaxillary fixation, intraocclusal block, kinesiotherapy, splint and only orientation.

The present findings should be cautiously interpreted. All included studies were retrospective and prospective, reducing the level of evidence because of the possible presence of uncontrolled confounding factors. Variables can not be isolated, such as: age, for example, younger children up to 10 years, will usually undergo non-surgical treatment, which can also be observed by the affected region, such as the mandible (more reported in this research), often undergoing non-surgical treatment, such as intermaxillary fixation.

Despite the difficulty of working with these patients further studies (preferably RCTs) with longer follow-up periods are recommended to investigate the most appropriate treatment for reducing facial fractures in children and adolescents.
